# Aggressive mediastinal amyloidosis mimicking malignancy: a case report

**DOI:** 10.1097/RC9.0000000000000925

**Published:** 2026-09-01

**Authors:** Sergio Andres Siado Guerrero, Jorge Ramírez Pereira, María Paula Motta Aguirre, Yeni Alexandra Arroyave-Guerrero

**Affiliations:** a Thoracic Surgery Fellow, Universidad El Bosque/ National Cancer Institute, Bogotá, Colombia; b Thoracic Surgeon, Clínica Marly, Hospital Santa Clara, Bogotá, Colombia; c Specialist in Bioethics, Universidad de La Sabana, Bogotá, Colombia; d Thoracic Surgeon, Universidad El Bosque/ National Cancer Institute, Bogotá, Colombia

**Keywords:** amyloidosis, case report, mediastinal mass, multiple myeloma, plasma cells

## Abstract

**Introduction:**

Amyloidosis is characterized by extracellular protein fibril deposition. Light-chain (AL) amyloidosis may be associated with plasma cell dyscrasias. Isolated mediastinal involvement is exceptionally rare and may mimic malignant tumors.

**Case presentation:**

A 69-year-old woman presented with a progressively enlarging anterior chest wall mass caused by an invasive mediastinal lesion. Biopsy revealed amyloid deposition. She underwent bilateral thoracotomy, sternectomy, *en-bloc* chest wall resection, and reconstruction. Final pathology confirmed AL amyloidosis associated with a kappa-restricted plasma cell neoplasm. Subsequent hematologic evaluation demonstrated active multiple myeloma.

**Clinical discussion:**

Mediastinal amyloidosis may present as a locally aggressive pseudotumoral mass mimicking malignancy. Histopathological confirmation is essential for diagnosis. The association between AL amyloidosis and plasma cell neoplasms may create additional diagnostic challenges and should be considered even in apparently localized thoracic presentations.

**Conclusion:**

This case highlights the diagnostic and therapeutic challenges of mediastinal AL amyloidosis with aggressive pseudotumoral behavior. Comprehensive hematologic evaluation should be performed early in all patients with localized thoracic AL amyloidosis to exclude occult plasma cell dyscrasia. Radical surgical resection may be justified in selected patients presenting with extensive local invasion and suspicion of primary thoracic malignancy. Long-term multidisciplinary surveillance remains essential because systemic progression may occur despite apparently localized initial disease.

## Introduction

Amyloidosis is characterized by extracellular deposition of protein fibrils, causing heterogeneous organ involvement^[^1^]^. Light-chain (AL) amyloidosis results from misfolding of monoclonal light chains produced by clonal plasma cells and is associated with plasma cell dyscrasias, including plasmacytoma and multiple myeloma^[^2,3^]^. Isolated mediastinal involvement is exceptionally rare and may mimic malignant tumors of the anterior mediastinum^[^4^]^. AL amyloidosis may be the initial manifestation of a plasma cell neoplasm or, less frequently, it presents as a localized form with potential systemic progression^[^2,5,6^]^

We report a 69-year-old woman with a large, invasive anterior mediastinal mass, initially suggestive of malignancy. Histopathological examination revealed AL amyloidosis associated with a kappa-restricted plasma cell neoplasm, and subsequent hematological staging demonstrated active multiple myeloma. This report was prepared in accordance with the SCARE criteria^[^7^]^

HIGHLIGHTS

- Mediastinal amyloidosis is an uncommon presentation that can radiologically mimic malignant tumors such as lymphoma or sarcoma.
- Histopathological examination was essential for diagnosis, revealing Congo-red-positive amyloid deposition associated with kappa-restricted plasma cell proliferation.
- Comprehensive hematologic evaluation uncovered an underlying plasma cell dyscrasia despite an initially localized thoracic presentation.
- Definitive management required extensive en-bloc resection with sternectomy and complex chest wall reconstruction.
- This case highlights the importance of thorough hematologic assessment and long-term surveillance in patients with thoracic amyloid masses.

## Case report

A 69-year-old woman with hypothyroidism and gastroesophageal reflux disease presented with a progressively enlarging anterior chest wall mass. Physical examination revealed a firm, nonmobile, 15-cm mass adherent to deep structures. Chest computed tomography (Fig. 1) demonstrated a calcified anterior mediastinal mass of size 129 × 113 × 180 mm causing leftward mediastinal displacement and sternal destruction. Differential diagnoses included sarcoma, germ cell tumor, and lymphoma; therefore, a thoracoscopic biopsy was performed.

**Figure 1.**
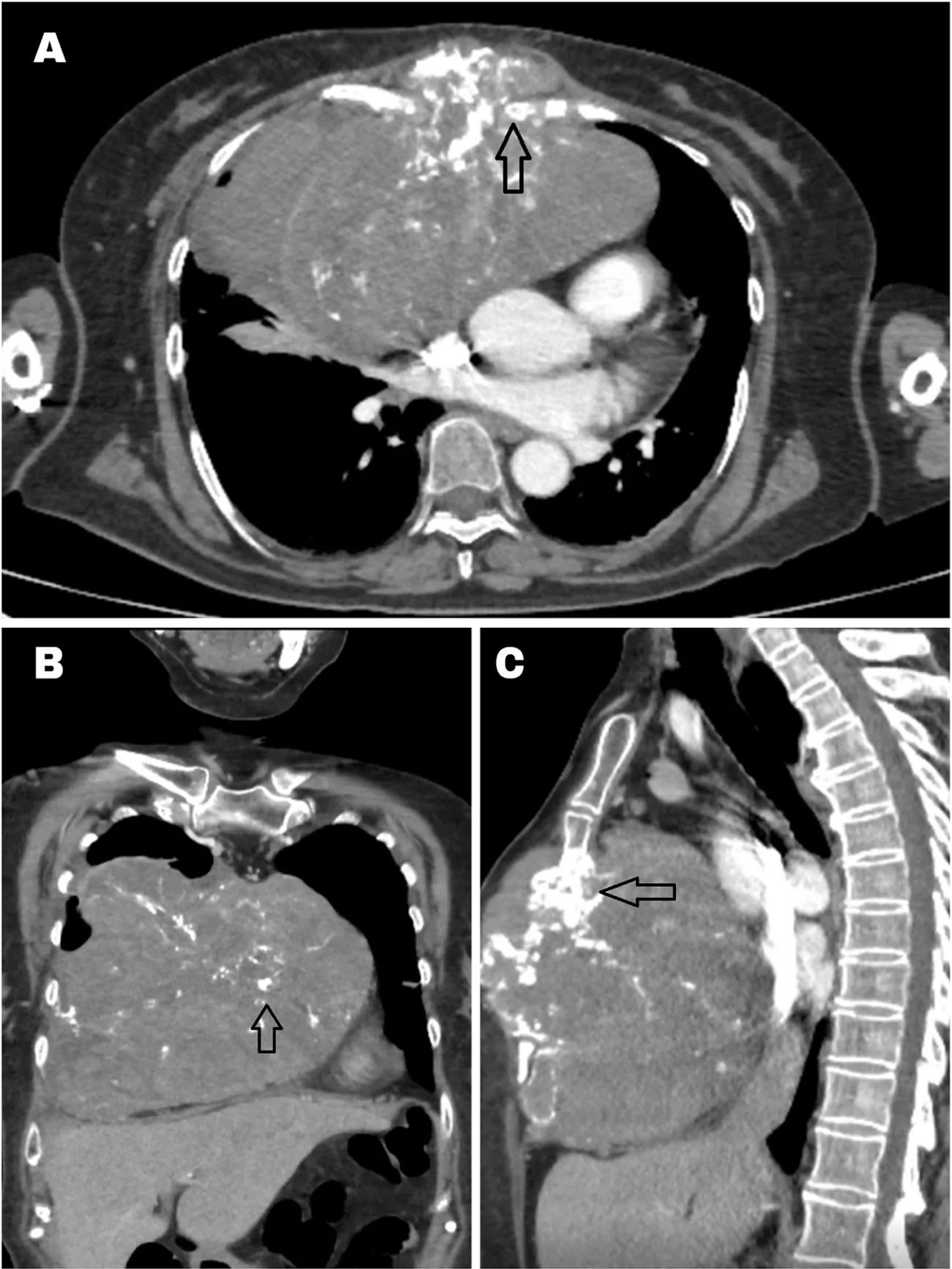
Preoperative chest computed tomography. (A) Axial view showing sternal involvement and internal calcifications, with leftward displacement of the heart and great vessels without evidence of invasion. (B) Coronal view showing a large anterior mediastinal mass with coarse internal calcifications measuring 129 × 113 × 180 mm (L × AP × T). (C) Sagittal view showing extensive anterior mediastinal involvement with posterior displacement of the great vessels and caudal displacement of the diaphragm.

Arrows indicate areas of calcification and sternal destruction.

Histopathological examination of the thoracoscopic biopsy specimens revealed lung parenchyma with hyaline material positive for Congo red staining, compatible with amyloid deposition, in addition to foreign-body–type granulomas. Despite these findings, progressive growth, sternal destruction, and chest wall invasion raised a persistent suspicion of malignancy. Following a multidisciplinary discussion, extensive resection and reconstruction were recommended.

The patient underwent bilateral thoracotomies with sternectomy, resection of involved skin, and *en bloc* resection of the anterior chest wall. This approach was selected because the tumor extended into both hemithoraces, thereby requiring wide exposure for complete resection and adequate reconstruction.

Chest wall reconstruction was performed using the STRATOS osteosynthesis system (MedXpert) in combination with a polypropylene synthetic mesh. Subsequently, soft tissue coverage was performed by the oncologic plastic surgery team using latissimus dorsi and pectoralis major muscle flaps (Figs 2 and 3). The surgery lasted approximately 9 hours, with an estimated blood loss of 500 mL. Perioperative antibiotic prophylaxis with cefazolin was administered.

**Figure 2.**
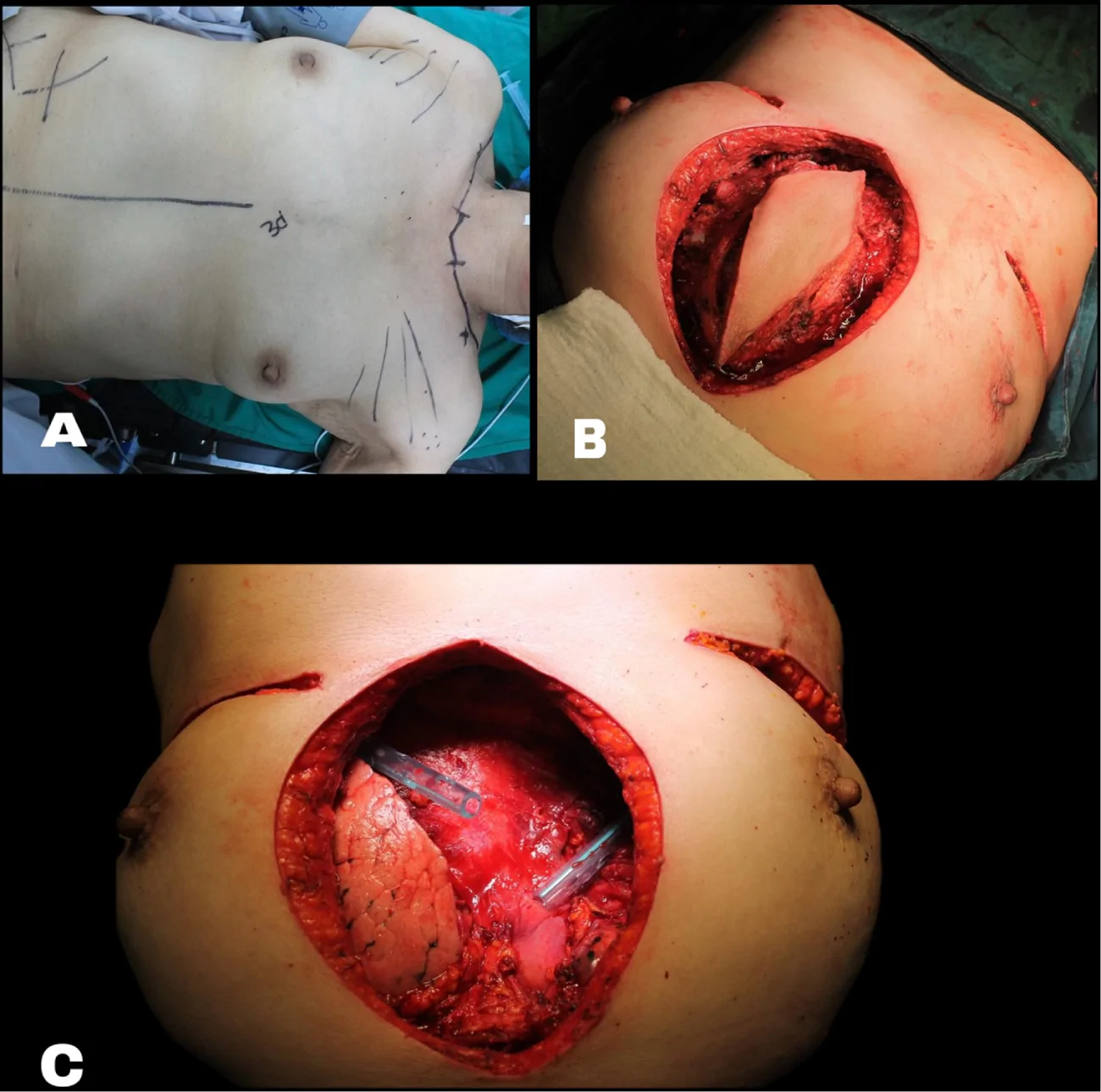
Surgical resection. (A) Preoperative photograph demonstrating surgical field marking for operative planning. (B) Intraoperative photograph showing resection of a skin ellipse overlying the lesion and bilateral costotomies of the second to seventh ribs, with dissection of mediastinal structures. (C) Anterior chest wall defect following resection of the mass, with the anterior mediastinum free of visible residual disease.

**Figure 3.**
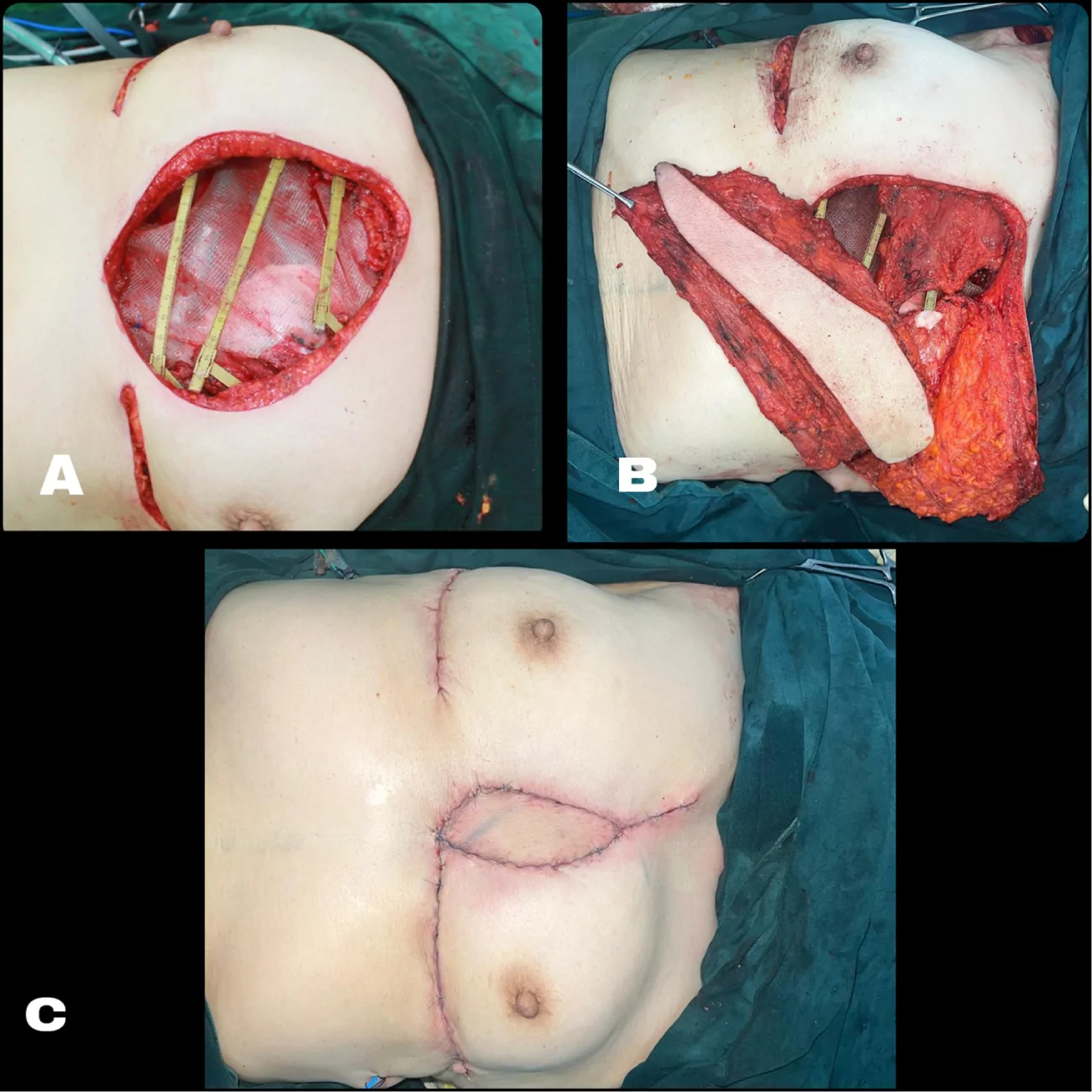
Chest wall reconstruction. (A) Chest wall reconstruction using mesh and osteosynthesis material to provide anterior thoracic wall stability. (B) Rotation of a fasciocutaneous flap to achieve adequate coverage of the anterior chest wall defect. (C) Final postoperative result.

Following surgery, the patient remained in the intensive care unit for 48 hours owing to the need for vasopressor support and was subsequently transferred to the ward after hemodynamic stabilization. Chest radiography showed adequate lung expansion and stability of the reconstructed chest wall. Chest tubes were removed on postoperative day 5. The patient was discharged on postoperative day 12 with two Jackson-Pratt drains, which were later removed. The postoperative course was favorable, and no complications were observed (no Clavien-Dindo–classified complications).

Definitive pathology demonstrated abundant amyloid deposition associated with atypical plasma cells. Immunohistochemistry showed positivity for CD38, CD138, CD56, and cyclin D1, with kappa light-chain restriction, supporting the diagnosis of AL amyloidosis associated with a kappa-restricted clonal plasma cell neoplasm; the Ki-67 proliferative index was 10%, and all surgical margins were negative.

Based on the pathology obtained from a thoracoscopic biopsy, a systemic disease evaluation was not initially performed. Following the definitive diagnosis, the patient was referred to Hematology. The systemic evaluation was completed, in a delayed manner, approximately 1 year after the initial diagnosis of AL amyloidosis due to delays in obtaining definitive histopathological results and scheduling additional studies.

Serum-free light chain analysis demonstrated a kappa-restricted monoclonal pattern, with kappa free light chains of 77.91 mg/L, lambda free light chains <0.66 mg/L, and a κ/λ ratio of 118. Serum protein electrophoresis did not demonstrate a discrete monoclonal spike, whereas urine protein electrophoresis revealed a monoclonal band in the beta region. Bence Jones protein testing was negative. Bone marrow biopsy, flow cytometry, and myelogram showed no evidence of plasma cell infiltration. Karyotype was normal (46,XX), and FISH testing was negative for the t(14;16) translocation. Transthoracic echocardiography showed no evidence of cardiac amyloid involvement. Positron emission tomography–computed tomography (PET-CT) demonstrated multiple hypermetabolic skeletal lesions consistent with plasma cell-related bone disease. In the context of a kappa-restricted clonal plasma cell neoplasm, a markedly abnormal serum free light chain ratio, and multifocal skeletal involvement on PET-CT, these findings fulfilled myeloma-defining (SLiM-CRAB) criteria for active multiple myeloma, and chemotherapy was initiated, followed by autologous stem cell transplantation.

After the diagnosis of active multiple myeloma, treatment with the CyBorD regimen (cyclophosphamide, bortezomib, and dexamethasone) was initiated. However, because of health care coverage limitations, the patient received only two treatment doses before therapy was interrupted. At the latest follow-up, 18 months after the initial presentation, the patient remained alive without evidence of local recurrence at the mediastinal surgical site (See Supplemental Digital Content Figure S1, available at: https://links.lww.com/IJSCR/A135).

## Discussion

The incidence of plasma cell disorders has increased in recent years, mainly due to population aging, with age as the main risk factor. The mean age at diagnosis of multiple myeloma is approximately 63 years^[^5,8^]^, which is consistent with our patient. Approximately 15% of patients with amyloidosis have clinically detectable amyloid deposits, and up to 25% of these cases are associated with kappa light chains; definitive diagnosis requires histopathological confirmation, including immunophenotyping and Congo red staining^[^5^]^.

Isolated mediastinal involvement by amyloidosis is an unusual presentation, with few cases reported in the literature^[^9,10^]^ and it may occur both in localized forms and within the spectrum of systemic plasma cell dyscrasias^[^4^]^. Imaging findings may show well-defined solitary or lobulated nodules with punctate calcifications^[^11–13^]^. Although amyloidosis is generally considered a benign infiltrative disorder, localized thoracic forms may occasionally present with pseudotumoral aggressive behavior, with invasion and destruction of adjacent structures^[^10^]^.

In our case, the patient presented with a large anterior mediastinal mass with coarse calcifications and sternal destruction, displacing mediastinal structures while sparing the heart and great vessels (Fig. 1). From a clinical and radiological standpoint, this presentation mimicked malignant lesions such as sarcoma or lymphoma, as previously described^[^9,10,14,15^]^. Although thoracoscopic biopsy was consistent with amyloid deposition, this aggressive presentation with suspicion of a primary malignant neoplasm supported the decision to perform extensive en bloc resection and complex chest wall reconstruction.

Definitive pathology confirmed AL amyloidosis associated with a kappa-restricted plasma cell neoplasm. This association may explain the observed pseudotumoral aggressive behavior and supports the need for comprehensive hematologic evaluation, even in apparently localized presentations.

Alternative management strategies should also be considered depending on the extent of the disease and the presence of systemic involvement. In patients with unresectable lesions or confirmed systemic amyloidosis, systemic chemotherapy directed at the underlying plasma cell clone represents the cornerstone of treatment, whereas surgery is generally reserved for diagnostic purposes, symptom control, or management of complications. Radiotherapy has also been reported for localized plasmacytomas or for residual disease in selected patients. In the present case, however, the extensive chest wall invasion, progressive sternal destruction, and persistent suspicion of primary thoracic malignancy justified radical resection followed by reconstruction.

Similar cases of chest wall and sternal plasmacytomas have rarely been reported. Msemo *et al* reported a giant, ulcerated, solitary plasmacytoma of the sternum requiring extensive resection and chest wall reconstruction^[^16^]^. Likewise, AbuGhannam *et al* described a paraosseous extramedullary thoracic plasmacytoma associated with multiple myeloma treated with systemic therapy^[^17^]^. Our case differs in the combination of a giant mediastinal mass with sternal destruction and AL amyloid deposits, requiring radical surgical management and complex chest wall reconstruction prior to the definitive diagnosis of systemic disease.

In this case, due to the strong initial suspicion of a primary chest wall or mediastinal neoplasm, a complete hematologic staging was not performed at the time of initial diagnosis. The systemic evaluation was performed in a delayed manner due to the delayed availability of definitive histopathological results and the scheduling of complementary studies. Therefore, it is not possible to determine with certainty whether the lesion represented a localized manifestation with subsequent progression or a systemic disease from the outset. This uncertainty reinforces the need for comprehensive hematologic evaluation and close follow-up in patients with AL amyloidosis associated with clonal plasma cell proliferations, given the heterogeneous behavior of these tumors and their potential progression to multiple myeloma^[^18,19^]^.

Although there are no universally standardized surveillance recommendations for patients with localized thoracic plasma cell neoplasms, the NCCN guidelines for solitary and extramedullary plasmacytomas recommend monitoring serum monoclonal protein levels every 3–6 months. FDG-PET/CT may also be useful for detecting recurrent or progressive disease^[^20^]^.

In complex and rare cases such as this, large language model–based artificial intelligence systems may facilitate the integration of complex clinical information and support risk stratification and individualized follow-up. However, these approaches still require clinical validation^[^21^]^.

This case highlights the diagnostic and therapeutic challenges of mediastinal amyloidosis with aggressive pseudotumoral behavior and suggests that some apparently localized thoracic lesions associated with amyloid deposits may represent an early or atypical manifestation within the spectrum of plasma cell dyscrasias. Therefore, comprehensive hematologic evaluation, multidisciplinary management, and prolonged surveillance are essential even in cases initially considered localized.

An important limitation of this case is the delayed systemic hematologic evaluation. Because complete hematologic staging was performed only after definitive surgical pathology, it remains impossible to determine whether systemic plasma cell disease was already present at the initial presentation or developed subsequently. Earlier assessment with serum free light-chain analysis, immunofixation electrophoresis, bone marrow biopsy, and whole-body imaging might have allowed earlier diagnosis and initiation of systemic therapy. This limitation underscores the importance of comprehensive hematologic evaluation in all patients diagnosed with localized AL amyloidosis, particularly when associated with plasma cell proliferation.

## Artificial intelligence statement

During the preparation of this manuscript, generative artificial intelligence (ChatGPT, OpenAI, GPT-5.5, cloud-based platform; July 2026) was used solely to assist in the graphical design of the clinical timeline (Supplemental Digital Content Figure S1, available at: https://links.lww.com/IJSCR/A135) during the manuscript revision process. Only de-identified clinical information describing the sequence of clinical events was provided as input. No identifiable patient information, diagnostic images, pathology slides, or confidential medical records were uploaded to the AI system.

All AI-generated graphical content was independently reviewed, verified, and edited by the authors to ensure complete consistency with the patient’s medical record and the final manuscript. Artificial intelligence was not used for study design, literature review, clinical interpretation, statistical analysis, or generation of scientific conclusions. The authors take full responsibility for the accuracy, integrity, and scientific content of this manuscript. No financial relationships or conflicts of interest exist with OpenAI or any AI software provider.

## Data Availability

This article reports a single clinical case. All data relevant to the case are presented within the manuscript. No additional datasets were generated or analyzed. Further information cannot be made publicly available due to patient confidentiality and privacy considerations.
